# Second course of re-irradiation in pediatric diffuse intrinsic pontine glioma: A case study

**DOI:** 10.1007/s00066-023-02057-x

**Published:** 2023-03-02

**Authors:** Paula Maria Bergengruen, Pablo Hernaíz Driever, Volker Budach, Daniel Zips, Arne Grün

**Affiliations:** 1 Department of Radiation Oncology, Charité—Universitätsmedizin Berlin, corporate member of Freie Universität Berlin, Humboldt-Universität zu Berlin, and Berlin Institute of Health, Berlin, Germany; 2 Department of Pediatric Oncology and Hematology, Charité—Universitätsmedizin Berlin, corporate member of Freie Universität Berlin, Humboldt-Universität zu Berlin, and Berlin Institute of Health, Berlin, Germany; 3 Department of Radiation Oncology, Charité—Universitätsmedizin Berlin, corporate member of Freie Universität Berlin, Humboldt-Universität zu Berlin, and Berlin Institute of Health, Campus Virchow-Klinikum, Augustenburger Platz 1, 13353 Berlin, Germany

**Keywords:** Childhood, Brainstem, High grade glioma, Progression, Radiotherapy

## Abstract

**Purpose:**

Concomitant chemoradiation followed by repeat (dose-deescalated) irradiation has become standard of care in treating childhood diffuse intrinsic pontine glioma (DIPG) during first line treatment and at first progression. Progression after re-irradiation (re-RT) is in most cases symptomatic and either treated systemically with chemotherapy or new innovative approaches including targeted therapy. Alternatively, the patient receives best supportive care. Data on second re-irradiation in DIPG patients with second progression and good performance status are sparse. This is a case report of second short-term re-irradiation to shed further light on this option.

**Methods:**

Retrospective case report of a 6-year-old boy with DIPG receiving a second course of re-irradiation (with 21.6 Gy) as part of an individual multimodal approach in a patient with very low symptom burden.

**Results:**

The second course of re-irradiation was feasible and well tolerated. No acute neurological symptoms or radiation-induced toxicity occurred. Overall survival was 24 months after initial diagnosis.

**Conclusion:**

A second course of re-irradiation can be an additional tool in patients with progressive disease after first- and second-line irradiation. It is unclear whether and to what extent it contributes to progression-free survival prolongation and if—since our patient was asymptomatic—progression-associated neurological deficits can be alleviated.

Diffuse intrinsic pontine glioma (DIPG) is a rare malignant central nervous system (CNS) tumor with a peak incidence at age 6 to 9 years [1]. Patients are usually diagnosed when neurological deficits like ataxia or bulbar palsy occur. Due to functional inoperability, standard treatment consists of definitive radiotherapy often combined with oral temozolomide (TMZ; based on established protocols in other malignant gliomas). Often, the clinical course is characterized by disease stabilization or partial remission eventually followed by local progression. Median overall survival in clinical trials is 7–16 months [2, 3]. Based on Vanan et al. (2015) [4] and Janssens et al. (2017) [5], the German childhood brain tumor network HIT recommends dose-deescalated re-irradiation for local progression ≥ 3 months after initial irradiation. In both analyses, re-RT was feasible and well tolerated, resulted in symptom relief in 77–90% of patients, was associated with a significant improvement in overall survival from 10.3 months (without re-irradiation) to 13.7 months [5], and an improvement in survival after progression from 95 days (historic cohort) to 171 days [4]. Further reports and reviews of a single cycle of re-irradiation in childhood DIPG have been published [6–8].

At the time of second progression, the question of a second course of re-RT seems obvious based on the aforementioned positive results. La Madrid et al. (2017) reported on 2 patients who received two courses of re-irradiation at 8 and 4–5 months and at 11 and 8 months, respectively, which were well tolerated [9]. The cumulative “equivalent dose in 2‑Gy fractions” (EQD2) at α/β2 (ratio of linear and quadratic components of cell kill typically proposed for CNS tissue) was 101 and 89 Gy, respectively. The patients died 4 and 12 months after the second re-irradiation.

## Methods

**Fig. 1:**
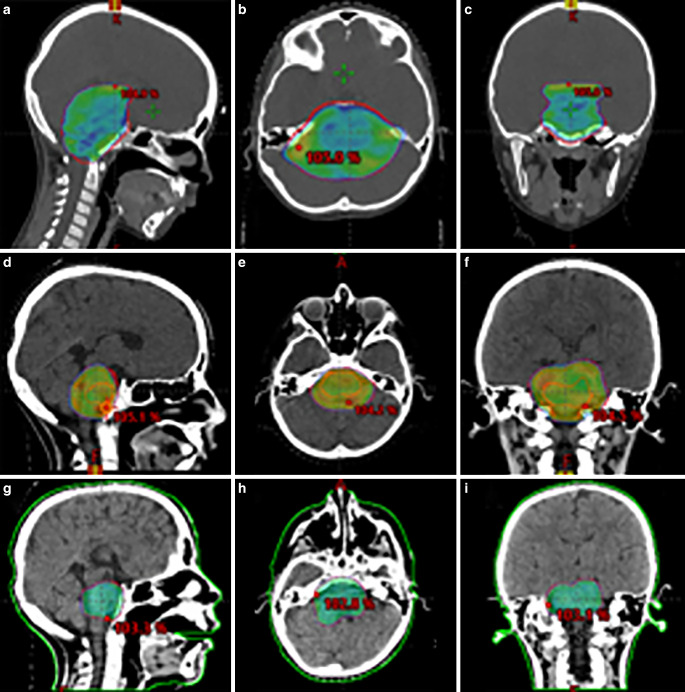
The three treatment plans in chronological order (from *top *to *bottom*) with representative slides (**a,d,g** sagittal; **b,e,h** axial; **c,f,i** frontal). *Color-wash* depiction of 95% of the prescription dose. The lateral cold spot in **b **was accepted with regard to cochlear sparing

This is a retrospective case report of an initially 6‑year-old boy with a radiologic diagnosis of DIPG (parents did not consent to biopsy). Evaluation of symptomatology and progression-free survival (PFS) was based on patient record/RT treatment plans/imaging. First irradiation was performed (based on the HIT-HGG 2013 study protocol) with CTV/PTV 2/0.5 cm to 54 Gy (1.8 Gy single dose [SD]) with concomitant TMZ followed by adjuvant TMZ and valproic acid. Re-RT (according to the 2017 HIT recommendation) was stereotactically guided with CTV/PTV 1/0.2 cm to 36 Gy (2 Gy SD) with concomitant and adjuvant VP16/trofosfamide (based on the HIT-REZ 97 study protocol). The second re-RT was stereotactically guided with CTV/PTV 0.5/0.2 cm to 21.6 Gy (1.8 Gy SD; Fig. 1). The resulting EQD2 to the brain and to the brainstem (without repair) was 117.8 Gy and 114.1 Gy (α/β2).

## Results

**Fig. 2:**
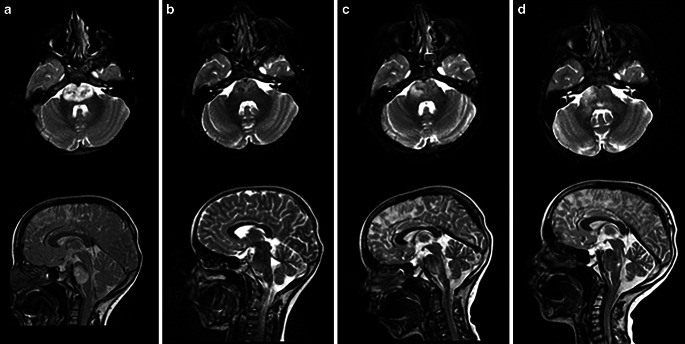
Imaging of the tumor in T2w-MRI axial and sagittal at the following timepoints: **a** initial diagnosis 01/2020, **b** 6 months after the initial irradiation, **c** progression 01/2021, **d** 1 month after the second re-irradiation

**Fig. 3:**
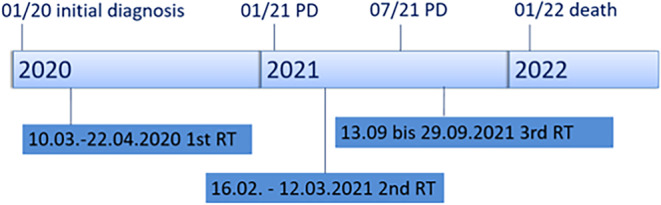
Timeline from initial diagnosis 01/20 via first progressive disease (*PD*) and second PD to death 01/22. *RT* ■■■

This is the case report of a 6-year-old boy with the incidental diagnosis of asymptomatic DIPG after traumatic brain injury in January 2020. Definitive radiochemotherapy started in March 2020 and was well tolerated. He received a total of 12 adjuvant cycles of TMZ. Follow-up MRIs at 1, 3, and 6 months showed partial remission followed by stable disease. Nine months after the end of RT there was radiographic but asymptomatic progression (Fig. 2c). Re-RT with concomitant VP16 (etoposide)/trofosfamide was again tolerated without neurological deficits. This resulted in a second partial remission. Six months after re-RT, radiographic and asymptomatic progression was noted. The patient still continued to show neither neurological deficits nor limitations of physical and mental performance when compared with peers. Due to the good general condition, our local tumor board decided to offer a second course of further de-escalated re-RT. Second re-RT was in September 2021 and tolerated without acute toxicity by the now 8.5-year-old patient. The first MRI in October 2021 showed possible progression/pseudo-progression (Fig. 2). Still, the patient remained asymptomatic. In December 2021, 3 months after re-re-RT, our patient developed progressive neurological symptoms including cranial nerve deficits (facial nerve palsy on the left, abducens nerve palsy on both sides), ataxia, and loss of strength and concentration. Systemic therapy was discontinued in January 2022 and palliative homecare was initiated. At this point, a total of nine cycles of oral trofosfamide/etoposide had been administered. He eventually succumbed to his disease shortly after, in late January. Time from re-re-irradiation to the patient’s demise was approximately 6 months, resulting in an overall survival of 24 months (Fig. 3).

## Discussion

DIPG is a malignant glioma of the brainstem with very poor prognosis. Concomitant radio- and chemotherapy as well as dose-deescalated re-RT have been established as a clinical standard in first-line therapy and at first progression. A second re-irradiation was described by la Madrid and coworkers in two cases and was well tolerated.

The represented case is exceptional due to the asymptomatic incidental finding, since DIPG is in most cases diagnosed when neurological deficits appear. As reported by la Madrid et al., irradiation was well tolerated in our case. Symptoms only appeared very late, with 3 months latency after the second course of re-irradiation. It is not clear whether the symptoms that occurred and subsequently led to the demise of the patient were solely due to progression of the underlying disease or at least in part a consequence of the treatment (i.e., necrosis).

On the one hand, one might argue that a lower initial irradiation dose might offer similar temporary symptom and disease control but would generate leeway in the form of dose reserve for re-irradiation, which is necessary since progression of the disease after irradiation is inevitable. On the other hand, it would be hard to argue in favor of such a strictly palliative approach in asymptomatic patients. Also, the impact of this concept alteration on PFS and OS is not clear.

## Conclusion

A second course of dose-deescalated re-irradiation may be considered in selected DIPG cases with patients in a good performance status at second progression after two courses of irradiation. The impact on longer-term tumor control and normal tissue effects needs to be evaluated in larger prospective cohorts. Due to the rarity of this constellation, we propose the initiation of a registry.
